# Narcissism as a protective factor against the risk of self-harming behaviors without suicidal intention in Borderline Personality Disorder. Preliminary results

**DOI:** 10.1192/j.eurpsy.2022.1707

**Published:** 2022-09-01

**Authors:** Í. Alberdi-Páramo, G. Montero-Hernández, M. Pérez Lombardo, J. Ibañez Vizoso, J. Pemán Rodríguez, L. Niell Galmés, J. Rodríguez Quijano, R.Á. Baena Mures

**Affiliations:** 1 Hospital Clínico San Carlos, Psychiatry, Madrid, Spain; 2 Hospital Fundación Alcorcón, Psychiatry, Madrid, Spain; 3 Hospital infanta Sofía, Psiquiatría, Alcobendas, Spain

**Keywords:** narcissism, protective factors against suicide, nonsuicidal self-injury, borderline personality disorder

## Abstract

**Introduction:**

The spectrum of suicidal behavior is a core factor of the prognosis and care of Borderline Personality Disorder (BPD).

**Objectives:**

Identify possible BPD specific personality traits that could act as protective factors of nonsuicidal self-injuries (NSSI).

**Methods:**

We performed a cross-sectional, observational and retrospective study of a sample of 134 BPD patients aged from 18 to 56. We assessed the presence or absence of suicidal behavior and NSSI as well as different sociodemographic variables. Millon, Zuckerman-Kuhlman and Structured Clinical Interview for DSM personality questionnaires were also applied. The analysis of the association between variables was carried out with a multivariate negative binomial logistic regression model.

**Results:**

A statistically significant association between NSSI and suicidal behavior was found. Elseways, statistically significant differences were also found in the association between NSSI and the SCID variables for Narcissistic Disorder, which appears as protective variables. These results provide an idea of the dynamic relationship between NSSI and suicidal behavior in a BPD population with particularly severe characteristics.

**Conclusions:**

The role of narcissistic personality traits appears to be important in identifying protective factors for NSSI and suicidal behavior in BPD patients and could be the subject of further research projects.

**Disclosure:**

No significant relationships.

